# Structural Insights Into Papain‐Derived Synthetic Antibacterial Peptides for Targeting *Klebsiella pneumoniae*

**DOI:** 10.1111/cbdd.70130

**Published:** 2025-05-23

**Authors:** Marcos Antônio Ferreira, Patrícia Souza e Silva, Adriel Parahyba Lacerda, Pedro de Mattos Franco, Fhillipe Ferreira Deodato da Silva, Thalis Ferreira de Souza, Maria Ligia R. Macedo, Ludovico Migliolo, Jefferson Soares de Oliveira

**Affiliations:** ^1^ Universidade Federal do Delta do Parnaíba – UFDPar Parnaíba Brazil; ^2^ S‐Inova Biotech, Programa de Pós‐Graduação em Biotecnologia Universidade Católica Dom Bosco Campo Grande MS Brazil; ^3^ Laboratório de Purificação de Proteínas e suas Funções Biológicas, Unidade de Tecnologia de Alimentos e da Saúde Pública Universidade Federal de Mato Grosso do Sul Campo Grande MS Brazil

**Keywords:** antimicrobial peptides, bacterial resistance, biofilms, enzyme model, molecular docking, papain

## Abstract

Bacterial resistance represents one of the greatest challenges in modern medicine, requiring innovative strategies. This study presents the rational design of two synthetic analogue peptides, WK‐MAP1, and WG‐MAP2, inspired by the structure of the enzyme papain (PDB 9PAP), emphasizing the novelty of using an enzyme as a model for developing new antimicrobials. Initially, in silico studies, including molecular modeling and docking experiments, revealed a high affinity of the peptides for mimetic bacterial membranes. Subsequently, in vitro assays confirmed their antimicrobial efficacy. WK‐MAP1 demonstrated superior activity against carbapenem‐resistant *Klebsiella pneumoniae* (KPC+), with a minimum inhibitory concentration (MIC) of 25 μM, whereas WG‐MAP2 exhibited activity against both tested strains (KPC+ and ATCC), with MICs of 50 and 100 μM, respectively. Both peptides effectively inhibited biofilm formation and exhibited low cytotoxicity in murine cells. This research highlights the potential of WK‐MAP1 and WG‐MAP2 as promising candidates for novel antimicrobial therapies, offering an innovative approach to overcoming the limitations of conventional antibiotics.

## Graphical abstract

The synthetic peptides WK‐MAP1 and WG‐MAP2, bioinspired by papain, demonstrated optimized activity, particularly against multidrug‐resistant *Klebsiella pneumoniae* , with improved sequence interaction profiles, membrane affinity, and predicted antimicrobial and antibiofilm efficacy, supporting their potential as therapeutic peptide candidates.

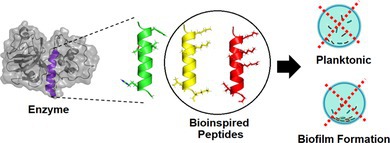

## 1 Introduction

The increasing prevalence of antibiotic resistance and biofilm formation in microbial infections has become a significant global health concern, necessitating the search for novel antimicrobial and antibiofilm agents. In this regard, brown algae from the Red Sea, particularly *Sargassum* sp., have garnered attention as a rich source of bioactive compounds with potential therapeutic applications (Alreshidi et al. 2023).

*Klebsiella pneumoniae* is a clinically significant Gram‐negative opportunistic pathogen responsible for various infections, including urinary tract infections, bacteremia, pneumonia, and liver abscesses (Wang et al. 2021). These infections can increase mortality rates in critically ill hospitalized patients and substantially raise hospital costs on a global scale (Ding et al. 2023). Due to its high level of antibiotic resistance, *K. pneumoniae* was included in the World Health Organization's list of priority pathogens in February 2017, being recognized as one of the greatest threats to public health (De Oliveira et al. 2020). The predominant resistance mechanism of this bacterium involves the inactivation of penicillins, as well as first‐, second‐, and third‐generation cephalosporins and aztreonam, through the hydrolysis of these antibiotics by the β‐lactamase enzyme (Hennequin and Robin 2016). Furthermore, *K. pneumoniae* exhibits a high capacity for biofilm formation, further complicating treatment.

Biofilm is a structured community of microorganisms embedded in an extracellular polymeric substance matrix (Versey et al. 2021). Its formation occurs in four stages: (1) reversible bacterial attachment, (2) adhesion and proliferation, (3) maturation, and (4) biofilm dispersion. Studies indicate that 75% of clinical *Klebsiella pneumoniae* strains form biofilm. Additionally, 97.1% of multidrug‐resistant (MDR) isolates exhibit this capability, with those originating from blood, pus, and tracheal secretions being the most prone to biofilm formation (Versey et al. 2021; Ashwath et al. 2022). The ability to form biofilm is a critical virulence factor of *K. pneumoniae* (Li and Ni 2023). A newly emerging hypervirulent strain of *K. pneumoniae* (hvKp) has raised concerns within the World Health Organization due to its high infection rate and resistance to the latest classes of antibiotics (De Oliveira et al. 2020).

Concurrently, the rising challenge of combating both antimicrobial resistance and cancer has driven the development of dual‐purpose drug candidates, highlighting the need for innovative strategies in drug discovery (Othman et al. 2021). In this context, antimicrobial peptides (AMPs) have emerged as promising candidates due to their unique mechanisms of action, which significantly differ from those of traditional antibiotics. This distinct feature makes AMPs highly attractive alternatives for addressing resistant pathogens (Giuliani et al. 2007). AMPs typically consist of 12–50 amino acid residues, characterized by their positive charges and amphipathic structures (Klousnitzer et al. 2024). They occur naturally in a wide range of organisms, including bacteria, plants, insects, and animals, and exhibit diverse therapeutic properties; plant‐derived AMPs have attracted considerable attention from the scientific and pharmaceutical communities due to their potential as models for the development of new antibiotic drugs (Gaur et al. 2018; Slezina et al. 2022). Furthermore, advances in chemical engineering have enabled the modification of AMPs to optimize key properties such as antimicrobial activity, target selectivity, solubility, and bioavailability (Riahifard et al. 2018; Falcigno et al. 2021). These modifications allow for the rational design of AMPs tailored to specific therapeutic needs.

Among natural bioactive compounds, papain, a cysteine protease derived from the latex of *Carica papaya* , has demonstrated notable antimicrobial properties (Juntavee et al. 2014). Studies reveal its efficacy against pathogens, such as *Staphylococcus aureus* , *Escherichia coli* , and *Pseudomonas aeruginosa* , as well as its applications in food technology, including the production of antimicrobial food packaging to prevent contamination (Mota et al. 2015). These attributes make papain a valuable molecule in the fight against microbial resistance.

However, the reliance on natural papain presents challenges, including seasonal variability in papaya availability and inconsistencies in purity and composition due to extraction processes. Synthetic peptides inspired by papain offer a promising solution to these limitations. Laboratory synthesis provides better control over production conditions, ensuring consistent quality, minimizing impurities, and enabling modifications to reduce allergenicity and side effects (Wu et al. 1998; Yazawa and Numata 2016). This approach not only circumvents the unpredictability of natural sources but also allows for the rational design of peptide analogues with enhanced antimicrobial properties.

Given these considerations, this study sought to design antibacterial peptides inspired by papain through rational approaches, such as molecular modeling and docking simulations. These advanced computational tools enable the identification of synthetic peptide analogues with enhanced efficacy, improved safety profiles, and greater potential to combat bacterial resistance. Such efforts aim to contribute to the development of next‐generation antimicrobial agents, addressing a critical global health challenge.

## 2 Methods

### 2.1 Rational Design of Peptide Analogues

The three‐dimensional structure of papain was used as a reference model to derive the peptide analogues in this study. Papain is a protease comprising a single polypeptide chain with 212 amino acids, organized into two distinct domains: the L domain, characterized by three α‐helices, and the R domain, which contains an antiparallel β‐sheet. The active site of papain resides in a cleft between these two domains, containing a cysteine residue essential for its catalytic activity (Kamphuis et al. 1984). The selection of the 18‐amino acid α‐helix WG‐18 (^26^WAFSAVVTIEGIIKIRTG^43^) as the study target was motivated by two fundamental characteristics. Firstly, the presence of the amino acid tryptophan at position 26 (Trp^26^) in this region is particularly significant due to its essential role in antimicrobial activity. Tryptophan is widely recognized as a critical component in AMPs because of its unique chemical properties, including its hydrophobicity and its ability to insert into and disrupt bacterial membranes. This property allows tryptophan to facilitate the interaction of AMPs with bacterial cell membranes, enhancing their efficacy in membrane permeabilization and cell lysis (Chan et al. 2006). Additionally, tryptophan's indole side chain provides a unique electrostatic interaction capability, which contributes to the peptide's selective activity against bacterial cells while minimizing toxicity to mammalian cells (Yang et al. 2024). Moreover, studies have shown that the positioning of tryptophan within the peptide sequence can further optimize its antimicrobial activity; for instance, peptides with tryptophan located near the N‐ or C‐terminal regions often demonstrate improved efficacy against resistant strains of fungi and bacteria (Kim et al. 2024).

The maintenance of the Trp^26^ residue in WG18 was aiming at the central dogma of biology. Therefore, the proposal was to alter the physicochemical characteristics to develop new peptides, termed multi‐activity peptides (MAPs), specifically WK‐MAP1 and WG‐MAP2. The modified peptide WK‐MAP1 underwent structural reorganization with point mutations at positions Phe^3^, Val^7^, Tyr^8^, Arg^16^, and Gly^18^, substituted by Lys^3^, Lys^7^, Ala^8^, Leu^16^, and Lys^18^, respectively, resulting in a sequence where the positive charge is solely contributed by lysine residues. Similarly, the WG‐MAP2 analogue was reorganized with substitutions at Phe^3^, Val^7^, Tyr^8^, Glu^10^, and Arg^16^, replaced by Lys^3^, Lys^7^, Ala^8^, Arg^10^, and Ile^16^, respectively, effectively repositioning arginine and glutamic acid within the sequence. The three‐dimensional structure of papain was retrieved from the Protein Data Bank (PDB) with the accession code 9PAP (Drenth et al. 1968). The structure model was predicted using the AlphaFold2 server and subsequently visualized with PyMOL 3.1 for analysis. The peptide sequences were analysed using the Schiffer‐Edmundson helical wheel projection via the HeliQuest server. Sequence alignments were performed using the Clustal Omega server version 1.2.4 to compare and calculate the percentages of identity and similarity between the native and modified sequences.

### 2.2 Validation of Peptides

To validate the generated models, two distinct steps were undertaken. Firstly, MolProbity was employed to assess the geometry of the peptide molecules, stereochemistry, and energy distribution, also calculating the mean score for dihedral angles while considering the covalent forces involved. In addition, QMEAN‐Swiss Model was utilized to compute the overall quality *Z*‐score of the models, ensuring that the scores fell within the acceptable ranges for native proteins. Tools such as CAMPr4 (support vector machine [SVM], random forest [RF] and artificial neural network), Sense the Moment (Machine Learning Algorithm), HemoPI 2.0 (SVM), and ToxinPred (SVM) were used to predict antimicrobial and hemolytic activities. These tools use hybrid models and machine learning algorithms to provide robust classification based on known database patterns.

### 2.3 Peptides Synthesis

The peptides were synthesized using the solid‐phase method with N‐9‐fluorenylmethoxycarbonyl (Fmoc) protection and subsequently purified by reverse‐phase high performance liquid chromatography (RP‐HPLC) to > 95% purity in a gradient of acetonitrile: H_2_O:TFA. The molecular mass of the peptides was determined using electrospray mass spectrometry conducted by Aminotech Company (São Paulo, Brazil).

### 2.4 Minimum Inhibitory Concentration (MIC) and Minimum Bactericidal Concentration (MBC)

The MIC assays were performed against *Klebsiella pneumoniae* (ATCC 13883) and *Klebsiella pneumoniae* (KPC + 001450421) strains. The bacteria were plated on Mueller‐Hinton agar (MHA) plates and incubated overnight at 37°C. After this period, three colonies of each bacterium were inoculated into 5 mL of Mueller‐Hinton broth (MHB) and incubated at 200 rpm, at 37°C, overnight. Bacterial growth was monitored at 600 nm using a spectrophotometer. The MIC assays were conducted using the microdilution method in 96‐well plates at a final bacterial concentration of 5 × 10^6^ CFU mL^−1^, as previously described (Wiegand et al. 2008). The peptides were tested at concentrations ranging from 6.25 to 100 μM. The antibiotic meropenem was used as a positive control at the same concentrations, whereas the bacterial suspension in MHB was used as a negative control. The microplates were incubated at 37°C for 18 h. After incubation, the microplates were read on a Multiskan Go microplate reader (Thermo Scientific) at 600 nm. MIC was determined as the lowest concentration of each fraction at which no significant bacterial growth was observed. For the MBC assays, 10 μL from each well where the MIC was determined were plated on MHA plates and incubated at 37°C for 24 h. MBC was defined as the lowest concentration of each fraction at which no bacterial growth was detected. Three independent experiments were performed for each condition tested.

### 2.5 Minimum Biofilm Inhibitory Concentration (MBIC)

Overnight cultures of *Klebsiella pneumoniae* (KPC + 001450421) were diluted to 5 × 10^6^ CFU.mL^−1^ in MHB medium and inoculated into Luria‐Bertani (LB) medium in 96‐well, round‐bottom cell culture microplates. The cultures were incubated in the presence of peptides at concentrations ranging from 6.25 to 100 μM for 24 h. The growth control contained only bacteria; Meropenem was used as a positive control for biofilm inhibition, whereas the negative growth controls contained only LB broth. After the incubation period, planktonic cells were removed, and the microplate wells were washed twice with deionized water. The remaining adherent bacteria were stained with 100 μL of 0.1% crystal violet (w:v) for 20 min. The microplates were washed twice with distilled water, air‐dried, and the biofilm was solubilized with 100 μL of 60% ethanol. The content was transferred to a new microplate, and the MBIC of the peptides was measured at 595 nm using a Multiskan Go microplate reader (Thermo Scientific). Three independent experiments were performed for each condition tested.

### 2.6 Hemolytic Assay

Hemolytic assays were performed with fresh blood from *Mus musculus* mice, which were collected and centrifuged to separate red blood cells and washed with 0.9% saline solution (pH 7.2). The peptide solutions were added to the erythrocyte suspension (1% by volume) at concentrations ranging from 6.25 to 100 μM. A 0.9% saline solution and Triton X‐100 were used as negative and positive controls, respectively. After exposure, hemoglobin release was measured at 415 nm (Park et al. 2004). All assays were performed in triplicate. This experiment was approved by the Ethics Committee on Animal Use of the Universidade Católica Dom Bosco under protocol number 014/2022. Three independent experiments were conducted for each condition tested.

### 2.7 MTT Assay

To evaluate the cytotoxicity of the peptides, a 3‐(4,5‐dimethyl thiazolyl‐2)‐2,5‐diphenyltetrazolium bromide (MTT) assay was conducted, following a modified protocol derived from the methodology described in the literature (Mosmann 1983). RAW 264.7 macrophage cell line and fibroblasts FN1 were maintained under sterile conditions according to established instructions. Cultured in DMEM, pH range of 7.2–7.4, (SIGMA) with 10% fetal bovine serum (SIGMA), the cells were incubated at 37°C in an environment of over 95% humidity and 5% CO_2_ within a 75 cm^2^ (KASVI) culture flask. Upon reaching 90% confluence, the cells were detached, centrifuged at 970 *g* for 5 min at room temperature, and then seeded at 2 × 10^5^ cells.well^−1^ in a 96‐well plate, according to Rezende et al. (2024); Buccini et al. (2018). Peptides at concentrations ranging from 6.25 to 100 μM were added and incubated at 37°C for 24 h. After discarding the supernatant, a (5 mg.mL^−1^) MTT solution (SIGMA) diluted in PBS was applied to each well. The covered plate was incubated for 4 h at 37°C in darkness. Formazan crystals were solubilized using a hydrochloric acid and isopropanol solution, and cell viability percentages were determined at 540 nm using a microplate reader (Thermo Scientific Multiskan). The results were calculated relative to the untreated cell control. The data were converted to percentage (%) according to the following equation:

$$\text{Cell viability}=\left(\frac{\mathrm{Abs}\;\text{sample}}{\mathrm{Abs}\;\text{positive control}}\right)\times 100$$

### 2.8 Circular Dichroism (CD) Spectrometry

CD analyses were performed using a Jasco J‐1100 spectropolarimeter (Jasco Inc., Japan) equipped with a quartz cuvette with a 1 mm path length. The spectrum from 185 to 250 nm was collected with a resolution step of 0.1 nm at 100 nm.s^−1^ at 25°C, averaging five accumulated scans for each condition. A 300 μM stock solution of peptides was prepared in water and used to create a 30 μM working solution. The secondary structure of the peptides was analysed in the presence of water, pH 7.0, 50% trifluoroethanol (TFE), pH 5.5, and 50 mM SDS, pH 7.5. The data were converted to molar ellipticity ([θ]) according to the following equation:

$$\left[\theta \right]=\frac{\theta }{10*C*1*\mathrm{nr}}$$

where θ is the measured ellipticity in millidegrees, C is the peptide concentration (M), l is the path length of the cuvette, and nr is the number of amino acid residues. The fractional α‐helix content (fH) was estimated using the equation:

$$\mathrm{fH}=\frac{\theta 222-\mathrm{\theta C}}{\mathrm{\theta H}-\mathrm{\theta C}}$$

where θC = 2220–53, and θH = (250 T—44,000) (1–3.n), with T representing temperature in Celsius, and n being the number of amino acid residues in the peptides. The values of θC and θH represent the limit values of mean ellipticity at 222 nm (θ_222_) for the disordered and α‐helix conformations, respectively. The helicity percentages of the peptides were predicted by the online tool, Bestsel with a scale factor of 3.5 (https://bestsel.elte.hu/index.php).

### 2.9 *Docking* Molecular

Membranes were constructed using the *Charmm GUI* membrane builder, using a ratio of 90% DPPE and 10% DPPG for gram‐negative membranes (Feng et al. 2020). *OpenBabel* software was used to prepare the receptor and peptides (O'Boyle et al. 2011). A grid box of 60 × 60 × 40 Å was defined for the top of the membranes, and 200 simulations were performed in *AutoDock Vina* (Trott and Olson 2010). The VinaAutomatic package was used to automate the simulations and subsequent analysis (available at https://github.com/mattospedrof/VinaAutomatic). For interaction analysis, the protein‐ligand interaction profiler web server was used (Adasme et al. 2021).

### 2.10 Statistical Analysis

The statistical significance of the experimental results was determined using a one‐way Student's *t*‐test or one‐way analysis of variance (ANOVA), followed by Dunnett's multiple comparison test. Values of *p* < 0.05 were considered statistically significant. GraphPad Prism version 8.0 was employed for all statistical analyses.

## 3 Results

### 3.1 Rational Design, Validation of Peptides and Synthesis

**FIGURE 1:**
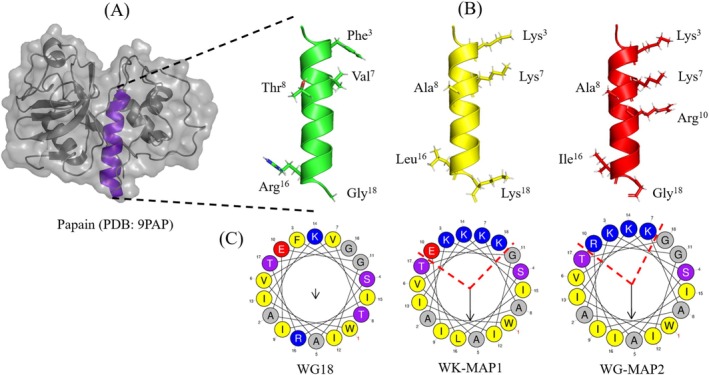
Three‐dimensional representation of the (A) papain enzyme (PDB: 9PAP) with the model portion used for designing the analogues highlighted in purple. (B) Three‐dimensional representation of the peptides. (C) Schiffer‐Edmundson helical wheel using HeliQuest, showing increased hydrophobic moments in the two analogues compared to the model α‐helix, represented by the arrows in the center. Hydrophobic faces are also represented by the yellow and gray amino acids at the bottom of the two analogues.

The three‐dimensional structure of papain (PDB 9PAP), specifically the segment from residues 26 to 43, was employed as a scaffold for the rational design of (Tables S1–S4) peptides (Figure 1A). This selected portion of the enzyme exhibits critical characteristics necessary for an AMP, including the presence of amphipathic α‐helices and residues such as tryptophan and arginine, which enhance interactions with bacterial membranes (Chan et al. 2006; Huang et al. 2010). The model peptide WG18 comprises 18 amino acid residues (^26^WAFSAVVTIEGIIKIRTG^43^) with a net charge of +1, a hydrophobic moment of 0.188, hydrophobicity of 67%, and a defined α‐helical structure. Following reorganization, the WK‐MAP1 peptide (WAKSAVKAIEGIIKILTK) displayed a net charge of +3, a hydrophobic moment of 0.638, hydrophobicity of 49%, and a defined α‐helical structure. Similarly, WG‐MAP2 (WAKSAVKAIRGIIKIITG) demonstrated a net charge of +4, a hydrophobic moment of 0.613, hydrophobicity of 53%, and a defined α‐helical structure (Figure 1B). Helical wheel plotting using HeliQuest revealed that WG18 lacked an organized distribution of polar and nonpolar amino acids. In contrast, the analogues showed a reorganization of polar and nonpolar residues on separate faces, imparting amphipathic properties to the peptides (Figure 1C). The analogue peptides maintained 72% identity with the model peptide WG18 (Table 1).

**TABLE 1:** Physicochemical characteristics of the native peptide and its analogs.

Name	Sequence	*Z*+	*Z*−	Z*	H	μH	NP (%)	ID (%)	MW (Da)
WG18	WAFSAVVTIEGIIKIRTG	+2	−1	+1	0.188	0.674	55.54	100	1961.3
WK‐MAP1	WAKSAVKAIEGIIKILTK	+4	−1	+3	0.638	0.496	55.56	72	1969.4
WG‐MAP2	WAKSAVKAIRGIIKIITG	+4	0	+4	0.613	0.536	55.56	72	1925.3

Abbreviations: ID, identify; MW, molecular weight; NP, nonpolar; Z*, net charge; *Z*−, negative charged; *Z*+, positive charged; μH, hydrophobicity moment; hydrophobicity.

To validate the three‐dimensional models generated by AlphaFold, the MolProbity software was used to generate Ramachandran plots. The analysis demonstrated that the phi (φ) and psi (ψ) torsion angles of the amino acid residues in WG18, WK‐MAP1, and WG‐MAP2 were predominantly located within allowed regions, with 98% corresponding to secondary conformations, particularly α‐helices. The QMEAN‐SwissModel tool was used to assess the reliability of AlphaFold‐generated models, providing *Z*‐scores of 0.67 ± 0.12 for WG18, 0.69 ± 0.12 for WK‐MAP1, and 0.59 ± 0.12 for WG‐MAP2, indicating varying levels of model reliability. The antimicrobial potential of the peptides was evaluated using artificial intelligence programs, as shown in Table S2. The model peptide WG18 showed limited antimicrobial and anticancer potential, with a low probability of being an AMP (0.677 in SVM). Conversely, WK‐MAP1 exhibited high antimicrobial potential across all classifiers, with a probability of 0.989 in SVM. Similarly, WG‐MAP2 demonstrated strong antimicrobial activity, achieving a probability of 0.975 in RF. None of the peptides showed significant cytotoxicity (Table 2).

**TABLE 2:** Functional predictions for WG18, WK‐MAP1, and WG‐MAP2 peptides based on the CAMPR4, Sense the Moment, HemoPI 2.0, and ToxinPred servers.

Peptide ID	CAMPr4			Sense the moment (ML)	HemoPI 2.0 SVM	ToxinPred SVM
	SVM	RF	ANN			
WG18	0.677	0.728	NAMP	0.1656	Hemolytic	−1.41
WK‐MAP1	0.989	0.9475	AMP	1.2510	Nonhemolytic	−1.38
WG‐MAP2	0.967	0.975	AMP	1.2434	Nonhemolytic	−1.10

*Note:* The parameters include prediction scores generated by machine learning classifiers: support vector machine (SVM), random forest (RF), and artificial neural network (ANN). Peptides are classified as AMP (antimicrobial peptide) or NAMP (nonantimicrobial peptide) according to each tool's criteria.

Additionally, the ToxinPred program predicted molecular masses consistent with synthesis values: 1960.30 Da for WK‐MAP1, 1968.40 Da for WG‐MAP2, and 1924.30 Da for WG18. These peptides were chemically synthesized with 95% purity, and their synthesized masses closely aligned with the predictions (Table 1). Both peptides were diluted in ultrapure water to prepare stock solutions and stored at −20°C for subsequent vitro assays. These validations and detailed analyses support the viability of these peptides as candidates for next‐generation antimicrobial agents.

### 3.2 Antibacterial Assays

**FIGURE 2:**
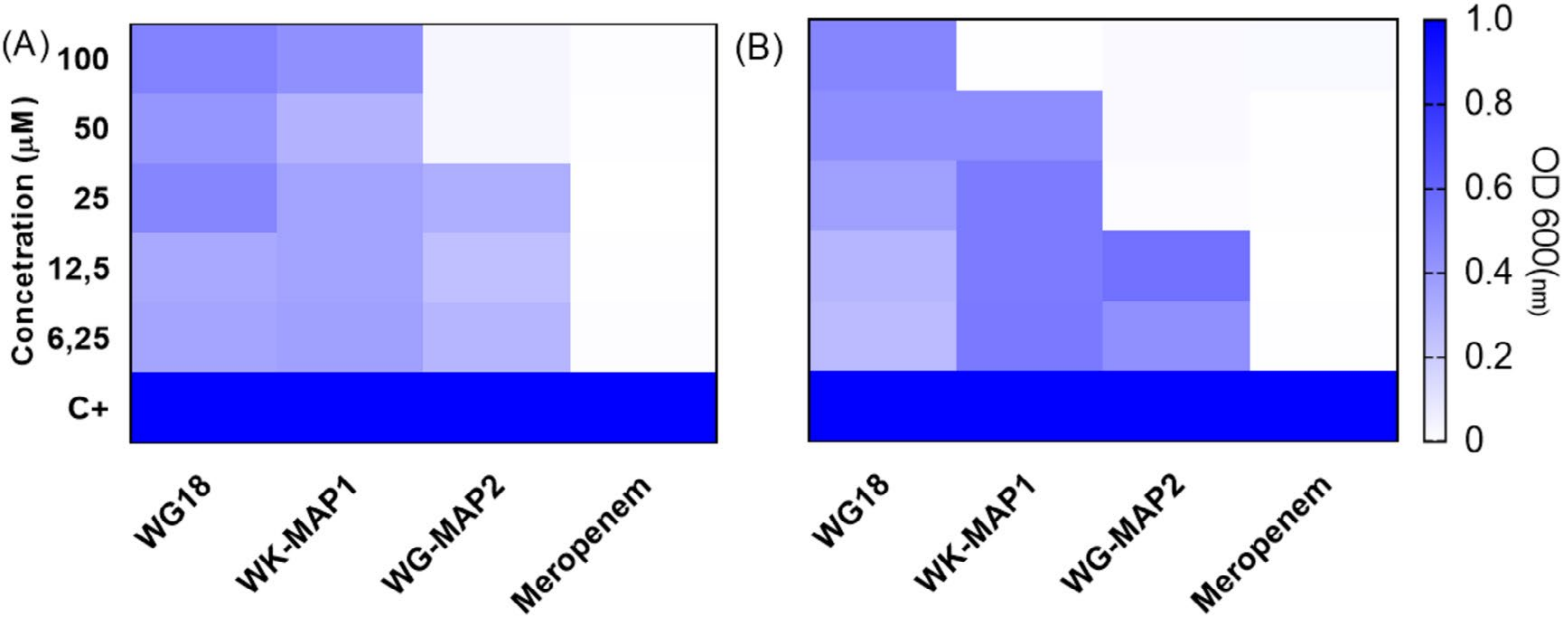
Minimal inhibitory concentration of (A) *Klebsiella pneumoniae* (ATCC 13883) and (B) *K. pneumoniae* (KPC + 001450421) for the peptides WG18, WK‐MAP1, and WG‐MAP2. Results are presented as optical density at OD_600 nm_ with values derived from experiments performed in triplicate. Statistical analysis was performed using two‐way ANOVA followed by Tukey test (****) *p* < 0.001.

The peptides WG18, WK‐MAP1, and WG‐MAP2 were evaluated against two *K. pneumoniae* strains: ATCC 13883 (a standard susceptible strain) and KPC + 001450421 (a carbapenem‐resistant, KPC‐producing strain). In vitro assays were performed using peptide concentrations ranging from 6.25 to 100 μM, with bacterial growth assessed relative to the untreated control (C+). The model peptide WG18 exhibited partial inhibitory activity at 100 μM against both strains. WK‐MAP1 showed partial inhibition against the ATCC strain and achieved a MIC of 100 μM against the KPC strain. WG‐MAP2 demonstrated inhibitory activity against both strains, with MIC values of 50 μM for the ATCC strain and 25 μM for the KPC strain (Figure 2). Additionally, MBC was observed only for WG‐MAP2, with values of 100 μM for the ATCC strain and 50 μM for the KPC strain (Figure S2).

### 3.3 Biofilm Formation Assays

The biofilm formation inhibition assay for the three peptides (WG18, WK‐MAP1, and WG‐MAP2) was performed against two *K. pneumoniae* strains: the ATCC reference strain and a KPC‐producing MDR strain. The peptides were tested at the same concentrations used in the MIC assays. The results are expressed as optical density at 595 nm (OD595) and transformed into percentage, indicating biofilm formation. WG18 showed no significant inhibitory effect on biofilm formation in the ATCC strain, even at the highest concentrations tested.

**FIGURE 3:**
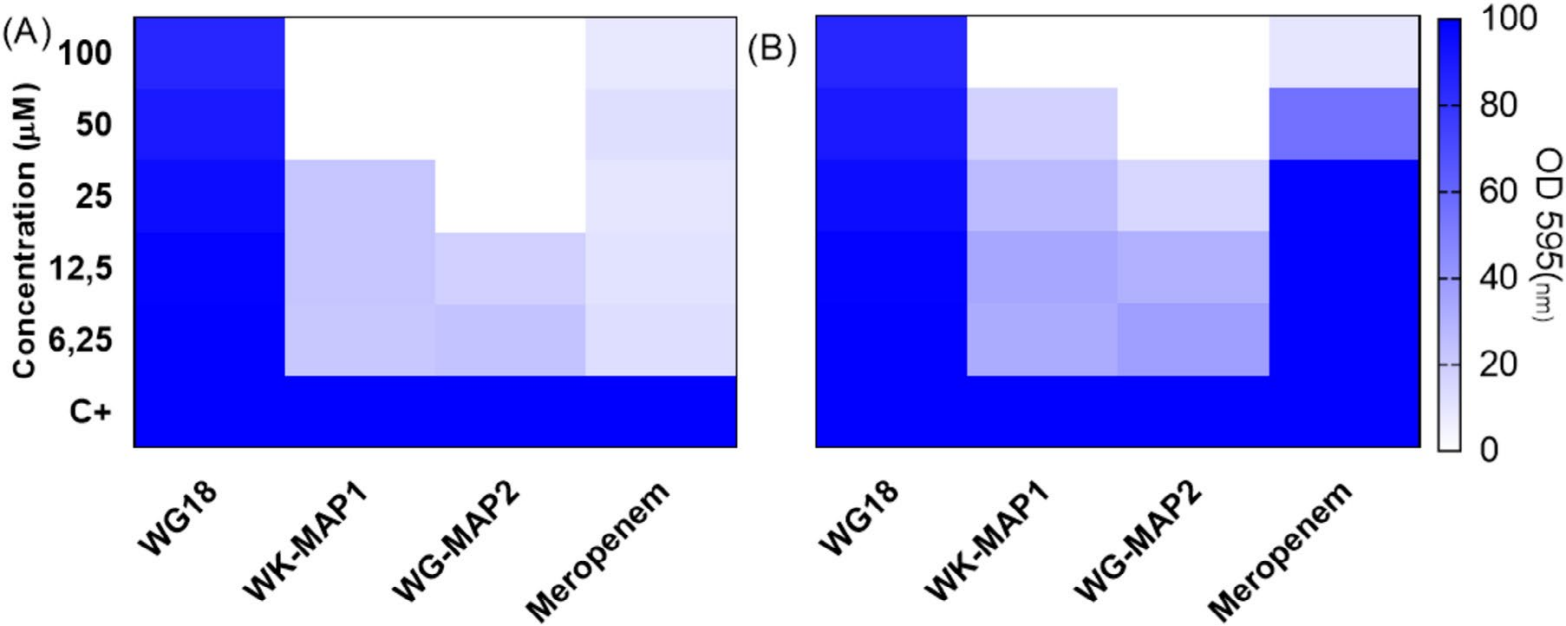
Minimal inhibitory concentration of biofilm formation of (A) *Klebsiella pneumoniae* (ATCC 13883) and (B) *K. pneumoniae* (KPC + 001450421) for the peptides WG18, WK‐MAP1, and WG‐MAP2. Results are performed in triplicate, and statistical analysis using two‐way ANOVA followed by Tukey test (****) *p* < 0.001.

In contrast, WK‐MAP1 significantly inhibited biofilm formation in the ATCC strain, with complete inhibition observed at 50 μM. WG‐MAP2 demonstrated a gradual reduction in biofilm formation at 25 μM. Similarly, WG18 exhibited no notable inhibition of biofilm formation in the KPC strain at any tested concentration. WK‐MAP1 was less effective against the KPC strain, achieving complete inhibition only at 100 μM. In the case of WG‐MAP2, there was inhibition of biofilm formation in the KPC strain at 50 μM, indicating effective disruption of biofilm development (Figure 3).

### 3.4 Cytotoxicity Activity

**FIGURE 4:**
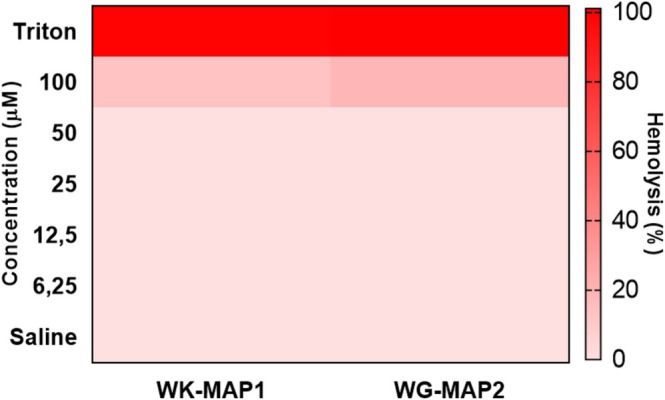
Hemolytic activity against murine erythrocytes for the peptide analogs WK‐MAP1 and WG‐MAP2 at different concentrations with values derived from experiments performed in triplicate. Statistical analysis was performed using two‐way ANOVA followed by Tukey test (****) *p* < 0.001.

The hemolytic activity of the peptides WK‐MAP1 and WG‐MAP2 was evaluated by measuring hemolysis percentages in murine erythrocytes. The results (Figure 4) demonstrate that both peptides exhibited low hemolytic activity, with significant effects observed only at the highest concentration tested (100 μM). For WK‐MAP1, hemolysis reached approximately 12%, whereas WG‐MAP2 caused around 18% hemolysis at 100 μM, indicating their relative safety for red blood cells at lower concentrations.

**FIGURE 5:**
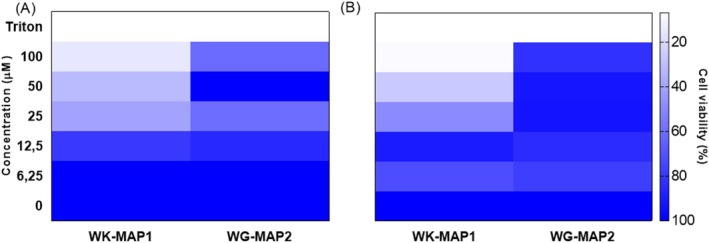
Cytotoxicity effect of the analog peptides WK‐MAP1 and WG‐MAP2 against cell line. (A) Cell viability of FN1 (human fibroblast) (B) cell viability of RAW (murine macrophages) with values derived from experiments performed in triplicate. Statistical analysis was performed using two‐way ANOVA followed by Tukey test (****) *p <* 0.001.

In addition to hemolytic activity, the cytotoxic effects were evaluated through cell viability assays in the FN1 and Raw cell lines. For FN1 cells, WK‐MAP1 also exhibited significant cytotoxicity in a dose‐dependent manner, with viability remaining around ~20%–25% at concentrations of 100 and 50 μM, respectively. However, its cytotoxicity decreased from 25 μM, where viable cells reached ~45%, ultimately achieving 100% viability at 6.25 μM. Lastly, WG‐MAP2 displayed moderate cytotoxicity against FN1 cells, maintaining ~50% cell viability at concentrations between 100 and 25 μM, with viability increasing to ~90% at 6.25 μM (Figure 5A). Regarding Raw cells, WK‐MAP1 significantly reduced Raw cell viability in a dose‐dependent manner. At 100 μM, the peptide resulted in approximately ~10% viable cells. Starting at 25 μM, the viability reached ~50%, and at 12.5 μM, the peptide lost most of its cytotoxicity, with viable cells increasing to ~90% (Figure 5B). In contrast, WG‐MAP2 had minimal impact on Raw cell viability, maintaining ~90% viable cells even at 100 μM.

The model peptide WG18 was not included in the cytotoxicity evaluations. This decision was based on the peptide's limited antimicrobial activity, as previously demonstrated in MIC and biofilm inhibition assays. Given its suboptimal performance in these critical tests, WG18 was deemed less relevant for cytotoxic analyses, as the primary focus of this study was on the optimized analogues, WK‐MAP1, and WG‐MAP2. These analogues were specifically designed to enhance antimicrobial efficacy and reduce cytotoxicity, making them the most promising candidates for further evaluation. This approach aligns with the study's objective to prioritize the exploration of analogues with improved therapeutic potential while using the model peptide WG18 solely as a reference scaffold. Such a strategy is commonly adopted in peptide design studies (Matalińska et al. 2020), where the focus is directed toward derivatives that exhibit superior performance.

### 3.5 CD Spectrometry

**FIGURE 6:**
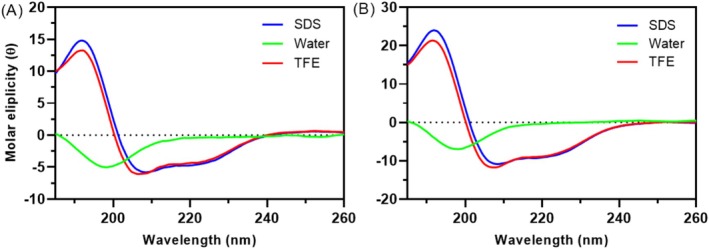
Conformational changes of synthetic peptides evaluated by circular dichroism in aqueous, TFE, and SDS environments. Spectra of (A) WK‐MAP1 and (B) WG‐MAP2 in water (green line), 50% TFE (red line), and 50 mM SDS (blue line). All spectra were collected at 25°C.

The conformational properties of WK‐MAP1 and WG‐MAP2 were analyzed using CD spectrometry under conditions simulating aqueous environments and anionic membranes (Figure 6). In an aqueous solution, WK‐MAP1 exhibited a random coil structure, characterized by a negative band between 195 and 208 nm and the absence of a signal near ~222 nm. Upon incubation with 50% TFE and 100 mM SDS, the CD spectrum of WK‐MAP1 underwent notable changes, showing a positive band around 192 nm and negative bands at 208 and 222 nm. These alterations suggest a structural transition indicative of α‐helix formation (Kelly and Price 2006). Similarly, WG‐MAP2 initially displayed a random coil conformation in an aqueous solution. However, when incubated with 50% TFE and 100 mM SDS, WG‐MAP2 exhibited a positive band near 192 nm and negative bands at 208 and 222 nm, indicating a conformational transition consistent with α‐helix structuring (Greenfield 2006).

### 3.6 Molecular Docking

**FIGURE 7:**
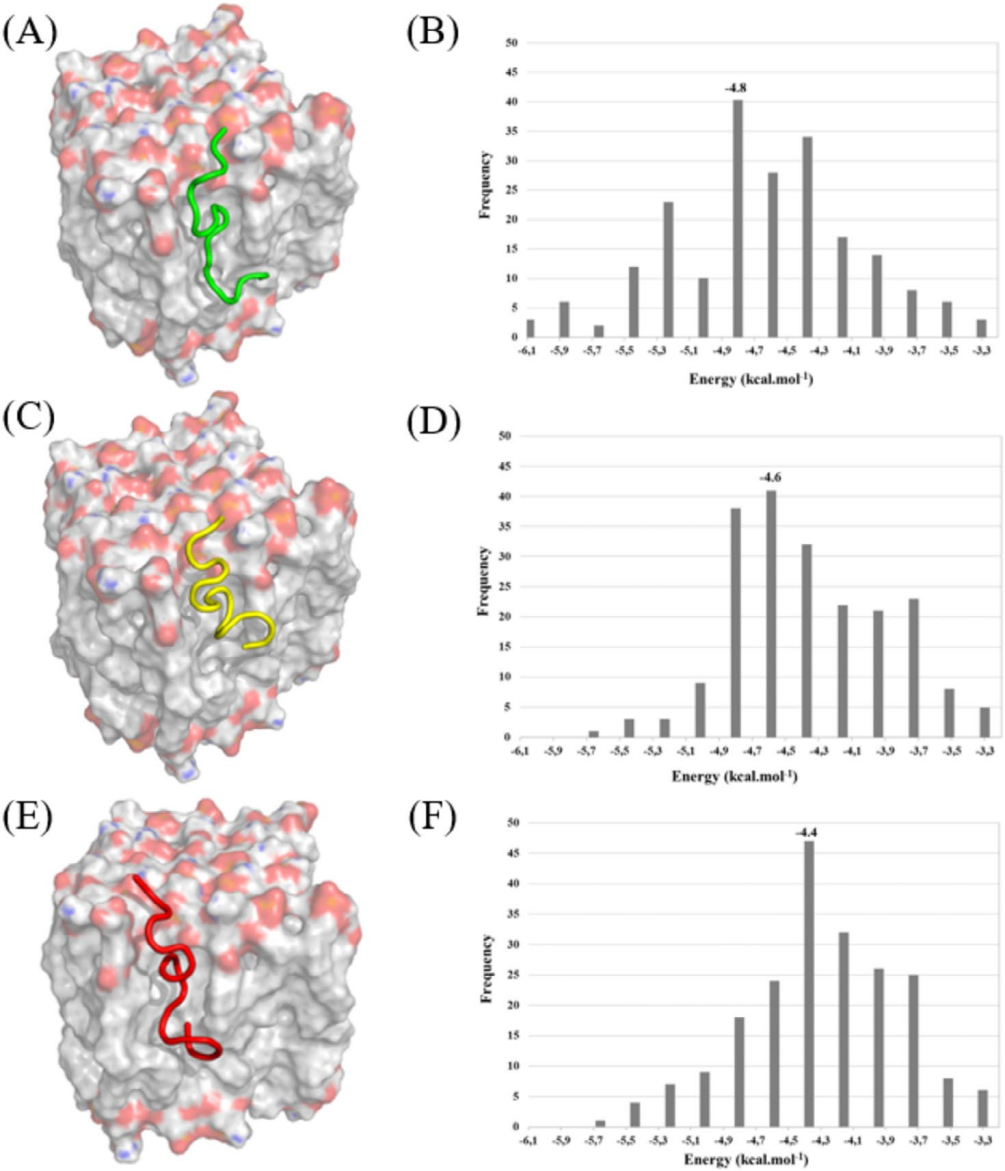
Molecular docking of the peptides interacting with gram‐negative phospholipid bacteria membranes. (A) WG18 (B) affinity energy frequency for the WG18 model (C) WK‐MAP1 (D) affinity energy frequency for the WK‐MAP1 model (E) WG‐MAP2 (F) affinity energy frequency for the WG‐MAP2 model.

Model selection for structural analysis was based on the frequency of interaction energies observed across 207 molecular dynamics simulations (Figure 7). The most frequently occurring binding energies for WG‐18, WK‐MAP1, and WG‐MAP2 were −4.8, −4.6, and −4.4 kcal·mol^−1^, respectively. These values correlate with the interaction profiles and in vitro findings. WG‐18 demonstrated affinity for the membrane mimetic, as indicated by the interaction data, yet this did not translate into significant antimicrobial activity. In contrast, WK‐MAP1 exhibited both membrane affinity and effective antimicrobial activity, particularly against KPC‐producing and biofilm‐forming bacteria. WG‐MAP2 not only showed strong membrane interaction but also yielded the most potent in vitro activity, with observed MIC values against both planktonic and biofilm‐forming ATCC and KPC strains.

Based on in silico results for the protein–ligand complex involving WG18 and a membrane mimetic model, multiple noncovalent interactions were identified, highlighting the peptide's binding affinity and specificity toward phospholipid components (Figure 7). WG18 exhibited several hydrophobic interactions with nonpolar residues, including tryptophan (Trp), alanine (Ala), phenylalanine (Phe), valine (Val), threonine (Thr), isoleucine (Ile), glutamate (Glu), arginine (Arg), and glycine (Gly). These interactions, with interatomic distances ranging from approximately 3.1–4.5 Å, suggest that the peptide is accommodated within a hydrophobic pocket, facilitating van der Waals contacts that enhance binding stability.

Two significant hydrogen bonds were observed: one with arginine (Arg) at a donor–acceptor distance of 3.7 Å and another with threonine (Thr) at 3.2 Å. These interactions, characterized by favorable donor angles, play a critical role in stabilizing the ligand orientation within the binding site and enhancing molecular specificity. In addition, one electrostatic interaction was detected between the ligand's carboxylate and the positively charged residues lysine (Lys), with a distance of 4.8 Å.

Similarly, the WK‐MAP1 analogue exhibited multiple noncovalent interactions, including hydrophobic contacts with Trp, Ala, Lys, Val, Ile, and Glu residues. These interactions, occurring at distances ranging from 2.9 to 4.0 Å, suggest that the peptide is likewise stabilized within a hydrophobic environment. Two prominent hydrogen bonds were identified with threonine residues at 3.0 and 4.0 Å, respectively, both contributing to accurate ligand orientation and increased binding specificity. Additionally, four electrostatic interactions involving lysine residues, with interatomic distances ranging from 3.6 to 4.3 Å, further supported membrane anchoring and overall complex stability.

Finally, the WG‐MAP2 peptide analogue established extensive hydrophobic interactions with nonpolar residues, including Trp, Ala, Val, Lys, Ile, and Arg, with interatomic distances ranging from 3.4 to 3.9 Å. Two significant hydrogen bonds were identified involving the amino acids Ile and Thr, acting as acceptor and donor, respectively, with distances of 3.2 and 3.1 Å. These interactions contribute to the correct orientation and stabilization of the ligand within the binding interface. Moreover, electrostatic interactions were observed involving a single arginine residue engaging in two interactions with the ligand DPP, with distances of 4.1 and 4.3 Å. These dual electrostatic contacts highlight the critical role of Arg in anchoring the peptide and promoting stable complex formation.

## 4 Discussion

The findings of this study highlight the potential of the peptides WK‐MAP1 and WG‐MAP2 as promising candidates for novel antimicrobial therapies. The importance of AMPs in addressing bacterial resistance is widely recognized, as outlined in the introduction of this research (Hetta et al. 2024; Erriah et al. 2025). These peptides provide a viable alternative to traditional antibiotics, particularly considering the alarming rise of MDR pathogens.

The helical wheel projections reveal a distinct amphipathic α‐helical structure in WK‐MAP1 and WG‐MAP2, characterized by a clear segregation of hydrophobic and positively charged residues. This amphipathicity is critical for membrane interaction and disruption, which likely enhances their antimicrobial and antibiofilm activities (Yeaman and Yount 2003). In contrast, WG18 displays a less pronounced amphipathic pattern, with a more scattered distribution of charged and hydrophobic residues, potentially resulting in reduced membrane affinity and lower bioactivity. Additionally, the presence of multiple lysine and arginine residues on one face of the helix in WK‐MAP1 and WG‐MAP2 may increase electrostatic interactions with negatively charged bacterial membranes, further promoting insertion and disruption. Therefore, the structural organization of side chains and the helical amphipathicity observed in WK‐MAP1 and WG‐MAP2 provide a rational explanation for their enhanced activity compared to WG18.

The α‐helical conformation observed through CD spectrometry emerged as an essential feature in the analogue peptides WK‐MAP1 and WG‐MAP2, demonstrating their potential to interact efficiently with bacterial membranes (Kelly and Price 2006; Greenfield 2006).

In antimicrobial assessments, WK‐MAP1 exhibited superior efficacy against *K. pneumoniae* KPC+, a MDR pathogen, with a MIC of 25 μM. Conversely, WG‐MAP2 demonstrated efficacy against both tested strains, ATCC and KPC+, with MIC values of 50 and 100 μM, respectively. These findings highlight the significance of amphipathic properties and positive charges in these peptides, which facilitate interactions with anionically charged bacterial membranes (Souza E Silva et al. 2022). Previous studies have shown that structural reorganization to enhance amphipathicity significantly improves antimicrobial activity (Batoni et al. 2016; Erriah et al. 2024).

The differential activity observed between the carbapenem‐sensitive *K. pneumoniae* ATCC 13883 and the carbapenem‐resistant KPC‐producing strain likely reflects structural and physiological adaptations associated with resistance (Yeaman and Yount 2003). Although carbapenemase production does not directly alter membrane phospholipid composition, it is often accompanied by decreased porin expression, increased efflux pump activity, and reduced outer membrane permeability, all of which contribute to diminished antimicrobial susceptibility (Pagès et al. 2008). Rational peptide modifications, such as increasing cationic charge or optimizing hydrophobicity and amphipathicity, can enhance activity against resistant strains by improving interactions with altered membranes. These findings support structure‐based optimization strategies to overcome resistance and enhance peptide efficacy across diverse bacterial phenotypes (Mahlapuu et al. 2016).

Meropenem was employed as the positive control in the biofilm inhibition assays due to its well‐documented efficacy against bacterial infections, including those associated with biofilm formation. This broad‐spectrum β‐lactam antibiotic, belonging to the carbapenem class, exhibits potent bactericidal activity by targeting penicillin‐binding proteins (PBPs), which are essential for the final stages of peptidoglycan cross‐linking. Inhibition of these PBPs disrupts cell wall synthesis, leading to structural instability, osmotic imbalance, and eventual cell lysis (Martinez et al. 2025). Its inclusion as a positive control is supported by previous studies demonstrating meropenem's activity in experimental models of biofilm formation, particularly involving pathogens such as *Pseudomonas aeruginosa* (Santosaningsih et al. 2024). *K. pneumoniae* , known for its strong biofilm‐forming capacity and increased antibiotic resistance, further justifies the use of meropenem as a benchmark compound to assess the biofilm‐inhibitory properties of novel agents in vitro (Ribera et al. 2019).

Interestingly, the amino acid tryptophan (Trp) is encoded by a single nonredundant codon (UGG) (Barik 2020). This unique encoding suggests that the presence of Trp in a protein or peptide is significant, as its biosynthesis incurs the highest cost of all amino acids (Barik 2020). Trp is classified as hydrophobic due to its uncharged side chain; however, it is not embedded in the hydrocarbon region of lipid bilayers. Instead, it is typically positioned toward the hydrophilic side (Mishra et al. 2018). The side chain of Trp may influence the lipid membrane, thereby contributing to the antibacterial activity of peptides (Feng et al. 2020). It is well established that for a peptide to exhibit potent antimicrobial activity, an increased positive charge and organized amphipathicity are essential for effective interaction with bacterial membranes. Additionally, a hydrophobicity of approximately 50% is critical to minimizing cytotoxicity (Silva et al. 2023).

A relevant finding was the biofilm inhibition activity exhibited by the analogue peptides. Although WG18 had no significant impact, WK‐MAP1 and WG‐MAP2 effectively reduced biofilm formation at concentrations above 50 μM, particularly against the ATCC strain. Biofilms represent a substantial clinical challenge, protecting bacteria from both antimicrobial agents and the host immune system (Roque‐Borda et al. 2025). The divergent trend observed between planktonic bacterial suspensions and biofilms is due to their distinct structural and physiological properties. Planktonic cells, being free‐floating and metabolically active, are more susceptible to antimicrobial agents, which is reflected in lower MIC values (Hall and Mah 2017). In contrast, biofilm‐forming bacteria are embedded in a protective extracellular matrix, exhibit reduced metabolic activity, and express resistance‐related genes, leading to significantly higher MBIC values. This difference highlights the need for both MIC and MBIC assays, especially in clinical settings involving chronic or device‐associated infections (Hall and Mah 2017).

Therefore, the observed divergence in antimicrobial susceptibility trends between planktonic and biofilm states underscores the necessity of employing both MIC and MBIC assays when evaluating antimicrobial efficacy, particularly in clinical contexts involving biofilm‐associated infections such as those linked to indwelling medical devices, chronic wounds, and mucosal surfaces. This distinction is critical for guiding effective therapeutic strategies and accurately interpreting in vitro antimicrobial testing outcomes. These results suggest that the designed peptides could play a pivotal role in preventing biofilm‐associated infections.

Regarding cytotoxicity, the analogue peptides exhibited low hemolytic activity in murine erythrocytes and limited cytotoxic effects in cell lines. The cytotoxic profiles of WK‐MAP1 and WG‐MAP2 peptides exhibited distinct behaviors across FN1 and RAW cell lines, highlighting their differential bioactivities and potential selectivity. WK‐MAP1 demonstrated potent cytotoxicity against FN1 cells in a dose‐dependent manner, with cell viability significantly reduced to approximately 25% at higher concentrations. This effect decreased progressively with lower concentrations, reaching full viability at 6.25 μM. Similarly, WK‐MAP1 induced substantial cytotoxicity in RAW cells, with only about 10% viability at 100 μM. Cytotoxicity decreased with concentration, showing approximately 50% cell viability at 25 μM and 90% at 12.5 μM, mirroring the pattern observed in FN1 cells. This suggests a broad‐spectrum effect amenable to therapeutic modulation via dose adjustment.

In contrast, Suchi et al. (2023) observed a moderately higher cytotoxic effect on RAW cells using 36 μM of the YS12 peptide from *Bacillus velezensis* , resulting in approximately 83% cell viability. These findings suggest that WG‐MAP2 is comparatively less cytotoxic to nontarget cells at equivalent or even higher concentrations. In addition, cytotoxicity assays with WG‐MAP2 showed approximately 90% and 50% viability in FN1 cells at concentrations of 6.25 and 25 μM, respectively. This analogue peptide exhibited minimal cytotoxicity in RAW cells, maintaining around 90% viability even at the highest tested concentration of 100 μM. Lay et al. (2019) described a peptide isolated from the *Nicotiana occidentalis* plant, which exhibited cytotoxic activity against human adult dermal fibroblasts at a concentration of approximately 10 μM further emphasizing the relative safety of WG‐MAP2 under comparable experimental conditions.

In correlation, the in vitro evaluation of WK‐MAP1 and WG‐MAP2 supports their distinct cytotoxic and hemolytic profiles. Although WK‐MAP1 displays potent and broad cytotoxicity, WG‐MAP2 exhibits selective action toward FN1 cells with low toxicity to RAW cells and erythrocytes. Comparative analysis with previously reported peptides underscores the advantageous profile of WG‐MAP2, suggesting its promise as a safer and more selective therapeutic candidate. Further studies involving mechanistic exploration and in vivo models are warranted to confirm its potential clinical applications.

Molecular docking experiments provided valuable insights into the mechanisms underlying the action of these peptides. The analogue peptides showed greater affinity for mimetic membranes of gram‐negative bacteria, efficiently penetrating these membranes. In biological membranes, hydrogen bonding primarily involves the polar headgroups of phospholipids. Functional groups such as phosphate ($-{\mathrm{PO}}_{4}^{3-}$) and hydroxyl (–OH), prevalent in phosphatidylethanolamine, phosphatidylglycerol, and cardiolipin. The main phospholipids in bacterial membranes act as key hydrogen bond donors and acceptors (Epand et al. 2016; Zhang and Rock 2008). These polar functional groups interact with peptide residues bearing amid, hydroxyl, or charged side chains, facilitating membrane recognition. Conversely, aliphatic residues like leucine, isoleucine, and valine are nonpolar and do not participate in conventional hydrogen bonding (White and Wimley 1999). Their primary role is hydrophobic interaction with the lipid acyl chains, contributing to membrane insertion and disruption, being the key features of AMP activity (Brogden 2005). Regarding gram‐positive models, we agree that their inclusion would offer valuable insight, given differences such as a thicker peptidoglycan layer and absence of an outer membrane (Silhavy et al. 2010; Brown et al. 2013). However, our focus on Gram‐negative membranes was guided by stronger in vitro activity against *K. pneumoniae* and corresponding in silico docking results showing higher interaction stability. Future studies will explore Gram‐positive systems to broaden our understanding of AMP selectivity. This observation aligns with previous findings that associate positive charge and amphipathic structure with the ability of AMPs to destabilize bacterial membranes (Hetta et al. 2024; Uchikoga et al. 2013).

Although AMPs present a promising alternative for the treatment of infectious diseases, their susceptibility to proteolytic degradation remains a significant limitation during their development (Lourenço et al. 2023). Given these challenges, there has been an increasing focus on exploring strategies to enhance the stability and delivery of AMPs, particularly through the use of nanostructures, to overcome potential limitations (Wang et al. 2021). AMPs offer a compelling alternative to conventional antibiotics in combating bacterial infections, as they tend to have a lower propensity to promote antimicrobial resistance compared to widely used antibiotics in clinical settings (Xuan et al. 2023). Furthermore, the validation of in vivo models is a critical component of drug development, as it enables the assessment of a complex biological system in relevant species, providing valuable insights into the pharmacokinetics, efficacy, and safety profiles of potential therapies (Pandey and Dvorakova 2019). Addressing the broader‐spectrum antimicrobial efficacy, stability, and delivery challenges of AMPs in such models is essential for advancing their therapeutic potential.

Although these results are promising, future research should explore the in vivo efficacy of these peptides and optimize their pharmacokinetic and pharmacodynamic properties. Overall, this study demonstrates the efficacy and safety of WK‐MAP1 and WG‐MAP2, solidifying their potential as candidates for innovative antimicrobial therapies. The rational design strategy employed, combined with comprehensive experimental evaluations, represents a significant advancement in the development of AMPs as a solution to bacterial resistance.

## 5 Conclusions

The findings of this study indicate that the peptides WK‐MAP1 and WG‐MAP2 hold promising potential as candidates for the development of novel antimicrobial therapies. These peptides demonstrated significant efficacy against MDR *K. pneumoniae* strains, with amphipathic properties and α‐helical conformations essential for efficient interaction with bacterial membranes. Furthermore, their ability to inhibit biofilm formation underscores their relevance as potential therapeutic tools in preventing device‐associated infections, which remain a major challenge in the clinical management of resistant pathogens.

To build on the progress achieved, further studies are required to evaluate their efficacy in in vivo models and to investigate their pharmacokinetic and pharmacodynamic properties. These steps are indispensable to translate the preliminary findings of this study into practical and safe clinical applications.

Further studies involving mechanistic exploration and in vivo models are warranted to confirm its potential clinical applications. In summary, this study represents a significant contribution to the development of AMPs as viable alternatives to address bacterial resistance, providing a solid foundation for future research and therapeutic innovations.

## Conflicts of Interest

The authors declare no conflicts of interest.

## Supporting information


Figure S1

Table S1

## Data Availability

The data that support the findings of this study are available on request from the corresponding author. The data are not publicly available due to privacy or ethical restrictions.
