# Supplementary material for: Structural Insights Into Papain‐Derived Synthetic Antibacterial Peptides for Targeting Klebsiella pneumoniae
Source: Chem Biol Drug Des. 2025 May 23;105(5):e70130. doi: 10.1111/cbdd.70130 (PMC12099485; doi:10.1111/cbdd.70130)
Supplement: Supplementary file 1 — Figure S1 [file CBDD-105-e70130-s001.docx]

Supplementary Material: **Structural Insights into Papain-Derived Synthetic Antibacterial Peptides for Targeting *Klebsiella pneumonia***

**
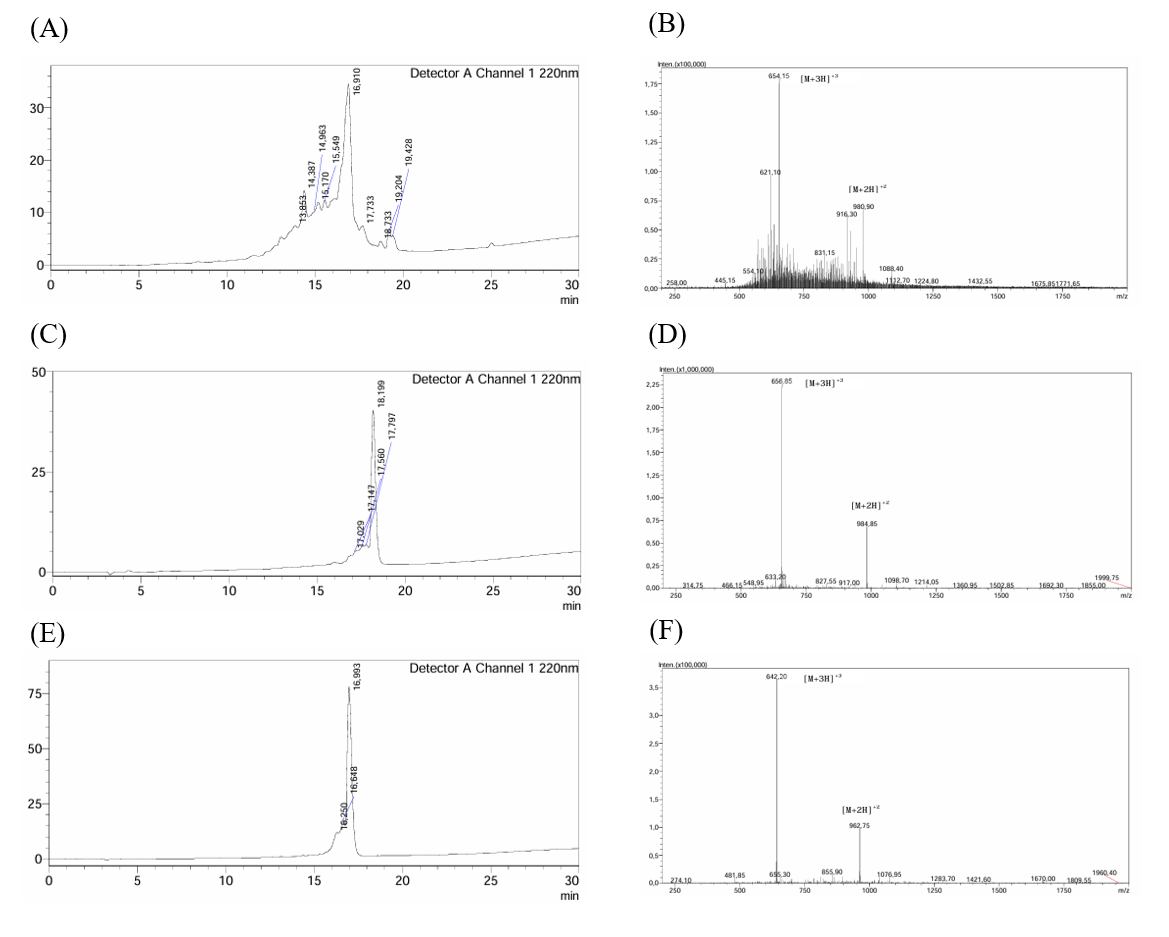
**

**Figure S1:** Purification by reversed-phase high performance liquid chromatography for (A) WG18 (C) WK-MAP1 and (E) WG-MAP2 with 95% purity. Electrospray mass spectra for (B) WG18 with 1961.3 Da (D) WK-MAP1 with 1969.4 Da and (F) WG-MAP2 with 1925.3 Da.

**
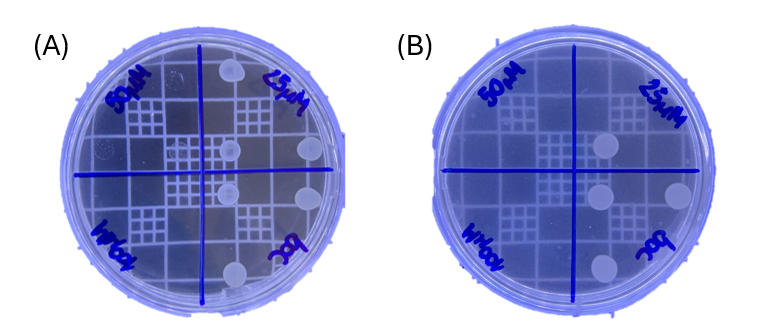
**

**Figure S2:** Minimal bactericidal concentration of (A) WG-MAP2 for the *K. pneumoniae* (ATCC 13883) and (B) WG-MAP2 for the *K. pneumoniae* (KPC+00145042**).**
