# Supplementary material for: Structural Insights Into Papain‐Derived Synthetic Antibacterial Peptides for Targeting Klebsiella pneumoniae
Source: Chem Biol Drug Des. 2025 May 23;105(5):e70130. doi: 10.1111/cbdd.70130 (PMC12099485; doi:10.1111/cbdd.70130)
Supplement: Supplementary file 2 — Table S1 [file CBDD-105-e70130-s002.docx]

Supplementary Material: **Structural Insights into Papain-Derived Synthetic Antibacterial Peptides for Targeting *Klebsiella pneumonia***

**Table S1:** Secondary structure by the BeStSel webserver for the analogue’s peptides WK-MAP1 and WG-MAP2 in different environments.

| **Environments** | **Conformation** | **Peptídes** | |
| --- | --- | --- | --- |
|  |  | **WK-MAP1 (%)** | **WG-MAP2 (%)** |
| **Water** | α-Helix | 0.0 | 0.0 |
|  | Turn | 13.0 | 8.6 |
|  | Other | 41.5 | 30.7 |
| **SDS 50 mM** | α-Helix | 61.5 | 93.5 |
|  | Turn | 0.0 | 0.0 |
|  | Other | 38.5 | 6.5 |
| **50% TFE** | α-Helix | 72.3 | 98.0 |
|  | Turn | 0.0 | 0.0 |
|  | Other | 27.7 | 2.0 |

**Table S2:** Atomic interaction distances between the WG18 peptides and DPPG phospholipid of Gram-negative bacterial membrane obtained from molecular docking analysis.

| Position | Residue | Protein Atom | Distance (Å) | Position | Phospholipd | Ligand Atom | Interaction Type |
| --- | --- | --- | --- | --- | --- | --- | --- |
| 1 | Trp | CZ2 | 3.7 | 41 | DPP | C37 | HI |
| 1 | Trp | CB | 3.6 | 43 | DPP | C311 | HI |
| 2 | Ala | CB | 3.8 | 18 | DPP | C310 | HI |
| 3 | Phe | CD1 | 3.6 | 18 | DPP | C312 | HI |
| 3 | Phe | CE1 | 3.5 | 18 | DPP | C311 | HI |
| 3 | Phe | CE2 | 3.6 | 18 | DPP | C39 | HI |
| 3 | Phe | CD1 | 3.6 | 43 | DPP | C316 | HI |
| 6 | Val | CG1 | 3.6 | 24 | DPP | C210 | HI |
| 7 | Val | CB | 3.6 | 41 | DPP | C315 | HI |
| 7 | Val | CG2 | 3.6 | 41 | DPP | C215 | HI |
| 7 | Val | CG1 | 3.7 | 41 | DPP | C313 | HI |
| 7 | Val | CG1 | 3.6 | 43 | DPP | C316 | HI |
| 7 | Val | CG1 | 3.6 | 9 | DPP | C313 | HI |
| 8 | Thr | CG2 | 3.8 | 24 | DPP | C28 | HI |
| 9 | Ile | CD1 | 3.7 | 18 | DPP | C311 | HI |
| 9 | Ile | CG1 | 3.8 | 9 | DPP | C312 | HI |
| 9 | Ile | CG1 | 3.4 | 9 | DPP | C212 | HI |
| 10 | Glu | CB | 3.4 | 9 | DPP | C32 | HI |
| 12 | Ile | CG2 | 3.8 | 24 | DPP | C29 | HI |
| 14 | Lys | O21 | 4.8 | 11 | DPP | Carboxylate | EI |
| 14 | Lys | O12 | 3.9 | 9 | DPP | Phosphate | HI |
| 16 | Arg | O | 3.7 | 18 | DPP | O14 | HB |

**Table S3:** Atomic interaction distances between the WK-MAP1 peptides and DPPG phospholipid of Gram-negative bacterial membrane obtained from molecular docking analysis.

| Position | Residue | Protein Atom | Distance (Å) | Position | Phospholipd | Ligand Atom | Interaction Type |
| --- | --- | --- | --- | --- | --- | --- | --- |
| 1 | Trp | CE3 | 3.6 | 18 | DPP | C312 | HI |
| 1 | Trp | CB | 3.7 | 18 | DPP | C210 | HI |
| 1 | Trp | CZ3 | 3.6 | 43 | DPP | HN1 | HI |
| 1 | Trp | CZ3 | 3.7 | 9 | DPP | C314 | HI |
| 2 | Ala | CB | 3.6 | 18 | DPP | C37 | HI |
| 3 | Lys | CB | 3.7 | 18 | DPP | C36 | HI |
| 3 | Lys | CD | 3.7 | 18 | DPP | C35 | HI |
| 6 | Val | CG2 | 3.8 | 24 | DPP | C29 | HI |
| 6 | Val | CG1 | 4.0 | 24 | DPP | HA | HI |
| 7 | Lys | CD | 3.9 | 18 | DPP | C312 | HI |
| 7 | Lys | CG | 3.4 | 9 | DPP | C314 | HI |
| 7 | Lys | CB | 3.8 | 9 | DPP | C212 | HI |
| 8 | Ala | CB | 3.6 | 9 | DPP | C34 | HI |
| 9 | Ile | CD1 | 3.5 | 11 | DPP | C36 | HI |
| 9 | Ile | CG2 | 3.9 | 11 | DPP | O32 | HI |
| 9 | Ile | CD1 | 3.8 | 11 | DPP | O32 | HI |
| 9 | Ile | CD1 | 3.6 | 9 | DPP | C311 | HI |
| 9 | Ile | CG1 | 4.0 | 9 | DPP | C38 | HI |
| 10 | Glu | OE2 | 2.9 | 9 | DPP | C33 | HI |
| 14 | Lys | Carboxylate | 3.6 | 18 | DPP | C32, C33 | EI |
| 14 | Lys | O | 3.6 | 9 | DPP | C21 | HI |
| 15 | Ile | CD1 | 3.6 | 9 | DPP | O32 | HI |

**Table S4:** Atomic interaction distances between the WG-MAP2 peptides and DPPG phospholipid of Gram-negative bacterial membrane obtained from molecular docking analysis.

| Position | Residue | Protein Atom | Distance (Å) | Position | Phospholipd | Ligand Atom | Interaction Type |
| --- | --- | --- | --- | --- | --- | --- | --- |
| 1 | Trp | CB | 3.4 | 18 | DPP | C311 | HI |
| 1 | Trp | CE3 | 3.8 | 18 | DPP | C37 | HI |
| 1 | Trp | CH2 | 3.6 | 18 | DPP | C26 | HI |
| 1 | Trp | CZ3 | 3.9 | 18 | DPP | C28 | HI |
| 1 | Trp | CZ3 | 3.8 | 19 | DPP | C215 | HI |
| 1 | Trp | CG | 3.6 | 9 | DPP | C313 | HI |
| 1 | Trp | CH2 | 3.6 | 9 | DPP | C211 | HI |
| 2 | Ala | CB | 3.9 | 41 | DPP | C214 | HI |
| 2 | Ala | CB | 3.7 | 43 | DPP | C316 | HI |
| 6 | Val | CG1 | 3.7 | 41 | DPP | C215 | HI |
| 7 | Lys | CB | 3.7 | 24 | DPP | C28 | HI |
| 7 | Lys | CD | 3.6 | 24 | DPP | C316 | HI |
| 8 | Ala | CB | 3.8 | 18 | DPP | C35 | HI |
| 9 | Ile | CG2 | 3.7 | 11 | DPP | C33 | HI |
| 9 | Ile | CD1 | 3.7 | 24 | DPP | C26 | HI |
| 9 | Ile | CD1 | 3.7 | 9 | DPP | C311 | HI |
| 9 | Ile | CG1 | 3.9 | 9 | DPP | C39 | HI |
| 9 | Ile | CG2 | 3.6 | 9 | DPP | C37 | HI |
| 10 | Arg | CB | 3.8 | 11 | DPP | C32 | HI |
| 10 | Arg | Phosphate | 4.3 | 24 | DPP | P | EI |
| 10 | Arg | Carboxylate | 4.1 | 24 | DPP | O22 | EI |
| 10 | Arg | CG | 3.5 | 9 | DPP | C32 | HI |
